# *In Vitro* Assessment of Injectable Bone Marrow Aspirate Concentrates Compared to Injectable Platelet-Rich Fibrin

**DOI:** 10.1007/s13770-024-00677-7

**Published:** 2024-11-04

**Authors:** Masako Fujioka-Kobayashi, Masateru Koyanagi, Ryo Inada, Ayako Miyasaka, Takafumi Satomi

**Affiliations:** 1 Department of Oral and Maxillofacial Surgery, School of Life Dentistry at Tokyo, The Nippon Dental University, 1-9-20, Fujimi, Chiyoda-ku, Tokyo, 102-8159 Japan; 2 Department of Oral and Maxillofacial Surgery, Faculty of Medicine, Shimane University, 89-1, Enya-cho, Izumo, Shimane 693-8501 Japan

**Keywords:** Platelet-rich fibrin, Platelets, Bone marrow, Cell culture

## Abstract

**BACKGROUND:**

Injectable platelet-rich fibrin (iPRF), a liquid form of PRF that is prepared from peripheral blood without anticoagulants, promotes tissue wound healing and regeneration. The present study focused on iPRF-like bone marrow aspirate concentrate (iBMAC) prepared without anticoagulant, and the regenerative potential of iPRF and iBMAC was compared *in vitro*.

**METHODS:**

iPRF and iBMAC were prepared from the same New Zealand white rabbits. The cytocompatibility and regenerative potential of each concentrate were evaluated using primary rabbit gingival fibroblasts and osteoblasts.

**RESULTS:**

Both gingival fibroblasts and osteoblasts treated with each concentrate exhibited excellent cell viability. Interestingly, compared to cells treated with iPRF, cells treated with iBMAC demonstrated significantly greater migration potential. Furthermore, higher mRNA levels of transforming growth factor-β (TGF-β), vascular endothelial growth factor (VEGF), and collagen I (COL1) were observed in gingival fibroblasts treated with iBMAC than in those treated with iPRF. Compared with osteoblasts treated with iPRF, osteoblasts treated with iBMAC exhibited greater differentiation potential, as indicated by increased osteocalcin (OCN) expression and mineralization capability.

**CONCLUSION:**

The results of the *in vitro* study suggest that, compared with iPRF, iBMAC may promote wound healing and bone regeneration more effectively. However, further preclinical and clinical studies are needed to confirm the regenerative potential of iBMAC in the body.

## Introduction

For the past thirty years, platelet-rich fibrin (PRF) has been employed in the field of regenerative medicine, with applications extending across a range of specialties, including dental medicine, maxillofacial surgical procedures, orthopedic interventions, and aesthetic reconstructive surgery [1–3]. The original PRF (leukocyte-PRF; L-PRF) forms from a solid (clot) when prepared in a glass tube or silica-coated plastic tube by a single centrifugation, whereas the liquid type of PRF, injectable PRF (iPRF), is developed by adjusting the centrifugation protocol and centrifugation tubes [4]. Blood centrifugation in nonglass (plastic) centrifugation tubes at lower centrifugation speeds results in iPRF [5]. iPRF is known for its numerous advantages over platelet-rich plasma (PRP), as iPRF is easy to handle, inexpensive and does not require an anticoagulant or bovine thrombin, which reduces biochemical alterations and risks associated with the use of bovine thrombin [4, 6]. Furthermore, similar to L-PRF, various growth factors are gradually released from iPRF continuously over 10–14 days [4]. In addition to these biological characteristics, recent studies have highlighted the antimicrobial, anti-inflammatory, and antibiofilm qualities of iPRF, and these studies have garnered interest in the literature [7].

Conventional bone marrow aspirate concentrate (BMAC), also referred to as marrow-derived PRP, has been developed, especially in the orthopedic field, to treat cartilaginous lesions, bone defects, and tendinous injuries [8, 9]. BMAC is generated using a centrifugation protocol similar to that used for PRP with anticoagulants, which yields hematopoietic stem cells, mesenchymal stem cells, and a concentration of growth factors; as a result, BMAC exhibits an elevated capacity to regenerate bones [8, 9]. The MSCs in BMAC might directly contribute to the repair of host tissue, and the nucleated cells could exert a paracrine influence, releasing various cytokines and growth factors to guide and enhance endogenous bone repair [10]. Centrifugation of bone marrow aspirates can amplify the cell concentration 6–7 times. This concentrated cellular content produces numerous growth factors, among which platelet-derived growth factor (PDGF), transforming growth factor-β (TGF-β), and vascular endothelial growth factor (VEGF) are likely the most significant [10].

Our group previously developed and investigated the possibility of using a solid form of BMAC (sBMAC) prepared without anticoagulants, similar to L-PRF [11, 12]. *In vitro* and *in vivo* studies revealed that, compared with PRF, sBMAC released more key growth factors, such as TGF-β, alkaline phosphatase (ALP), and osteocalcin (OCN), within 24 h and could serve as a more potent alternative to PRF for skin and bone regeneration, as well as conventional BMAC [11, 12]. However, the possibility of using injectable BMACs fabricated without anticoagulants (iBMACs) for tissue regeneration has not been determined. It was hypothesized that, similar to conventional BMAC, iBMAC could be easily handled, biocompatible and reliable for tissue regeneration.

Therefore, the present study utilized peripheral venous blood and bone marrow aspirates from a homogeneous population of rabbits to compare the handling procedures between the same individuals. iPRF and iBMAC were generated via a standardized centrifugation protocol. Furthermore, comprehensive evaluations of cellular dynamics—including parameters such as cellular viability, migration potential, gene expression markers, collagen synthesis, and mineralization potential—have been conducted *in vitro* on rabbit-derived gingival fibroblasts and osteoblasts treated with either PRF or iBMAC.

## Materials and methods

### Preparation of iPRF and iBMAC

Five New Zealand white male rabbits (3.5–4.0 kg; Nippon Bio-Supp. Center, Tokyo, Japan) were utilized to obtain blood/bone marrow concentrates and primary culture cells in the present study. All the animals were treated without excessive or disruptive noise and were fed a standard diet and water ad libitum at the Central Animal Care Facility at XXX University. This study was approved by the Animal Experiment Ethics Committee of XXX University (Approved No. 20-08-01).

**Fig. 1:**
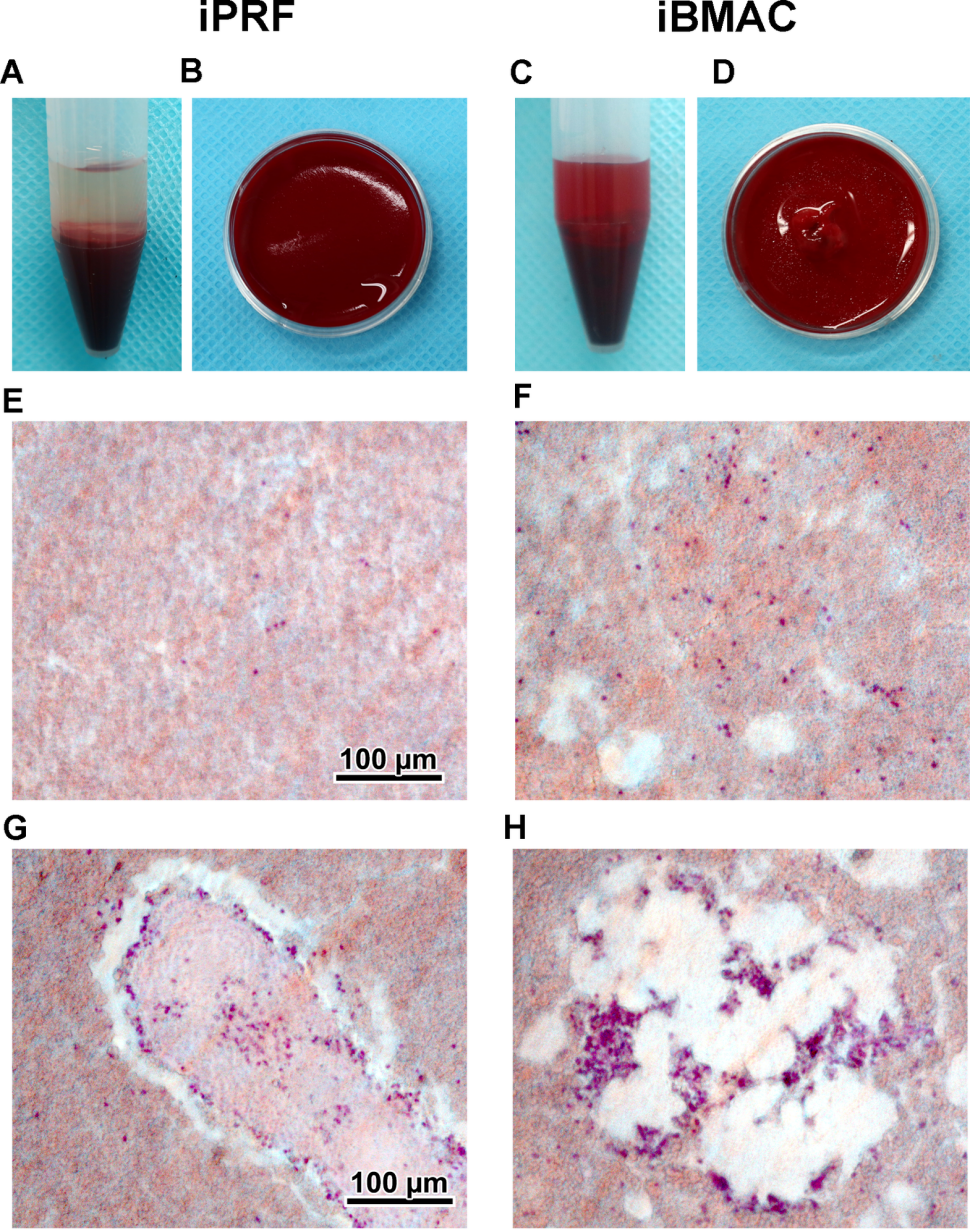
**A**–**D** Visual representation of layer separation of injectable platelet-rich fibrin (iPRF) or injectable bone marrow aspirate concentrate (iBMAC). (**A**, **C**) The separated layers after centrifugation of blood/bone marrow aspirate. (**B**, **D**) The iPRF or iBMAC generated from cell culture plastics. **E**–**H** Histological observation of frozen sections of each concentrate material. (**E**, **F**) The general parts of iPRF and iBMAC. More hematoxylin-stained nucleated cells were observed in the iBMAC specimens than in the iPRF specimens. (**G**) Aggregated clusters of cells containing leukocytes were occasionally observed in the iPRF specimens. (**H**) Many hematoxylin-stained cells, including leukocytes, adipocytes, and other bone marrow stromal cells, were located within and around the cluster of platelets and adipose cells in iBMAC

General anesthesia was applied by intramuscular injection of a mixture of butorphanol tartrate (Meiji Seika Pharma Co., Ltd., Tokyo, Japan), medetomidine hydrochloride (Nippon Zenyaku Kogyo Co., Ltd., Fukushima Japan), and midazolam (Astellas Pharma, Inc., Tokyo, Japan). Arterial blood was collected from the auricular arteries, and bone marrow aspirate was collected from the iliac bones. Each 2.5-mL blood or bone marrow aspirate was centrifuged in a plastic tube without anticoagulant at 700×*g* for 5 min at room temperature in a horizontal centrifuge machine (LC-200, TOMY, Tokyo, Japan). After centrifugation, the upper layer of components with the buffy-coat layer site and an approximately 5-mm red blood cell layer were collected as iPRF or iBMAC (Fig. 1A, C). The 5-mm red blood cell layer includes more cellular components releasing growth factors [13, 14] and this fraction should be included for clinical use [15].

### Histological sample preparation

The collected iPRF and iBMAC cells were transferred to cell culture plastic dishes and clotted within 10 min (Fig. 1B, D). The clotted specimens were fixed in 4% formaldehyde (Nacalai Tesque, Kyoto, Japan) in PBS and embedded in supercryoembedding medium (SCEM, Section-Lab, Hiroshima, Japan). The frozen specimens were sectioned into 8-μm-thick slices in the chamber of a cryomicrotome (Leica CM3050S, Leica, Tokyo, Japan) with adhesive film (Cryofilm Type 2C (16UF), Section-Lab) as previously reported [11, 16]. Images of the sections stained with hematoxylin were captured with an AXIO Imager M2 microscope (Carl Zeiss, Jena, Germany).

### Scanning electron microscopy (SEM)

The prepared iPRF and iBMAC were fixed overnight at 4 °C using a 2.5% glutaraldehyde solution (Sigma Aldrich, St. Louis, MO, USA), dehydrated in an elevated ethanol series and dried with hexamethyldisilazane (Sigma Aldrich). The dried specimens were attached to aluminum stubs (Nisshin-EM, Tokyo, Japan) and sputter-coated with platinum with a sputtering device (E-1030 Ion Sputter, HITACHI, Tokyo, Japan). SEM images were acquired using a scanning electron microscope (SEM; JSM-IT200, JEOL, Tokyo, Japan).

### Cell culture

Either iPRF or iBMAC was incubated in 10 mL of DMEM (Gibco, Life Technologies, Carlsbad, CA, USA) supplemented with 1% antibiotics (Gibco) at 37 °C. After 24 h, the incubated medium (conditioned medium) was collected and stored at − 20 °C until use. When added to the culture medium, both iPRF and iBMAC gradually coagulated within 10–15 min in the media. Therefore, the conditioned media did not contain the iPRF or iBMAC in their original forms; rather, they contained the growth factors released from these materials.

Rabbit primary gingival fibroblasts (RGFs) and rabbit primary osteoblasts (ROBs) were isolated and cultured from gingival tissue and bone chips from the mandible of rabbits, respectively, as previously described [11]. Since rabbits were selected as the source of the blood and bone marrow in the present study, rabbit gingival fibroblasts (RGFs) and osteoblasts (ROBs) were isolated to test the effects of iPRF and iBMAC from the same animals. Both cell lines were cultured in DMEM supplemented with 10% fetal bovine serum (FBS; Gibco) and 1% antibiotics in cell culture flasks at 37 °C in a humidified atmosphere. The cells were detached from the tissue culture plastic with 0.25% EDTA-trypsin (Gibco) before reaching confluency. The cells from passages 4–6 were used for experimental cell seeding.

The cells were seeded in medium supplemented with 20% conditioned medium (or 20% control medium without FBS) and 80% basic cell culture medium (DMEM supplemented with 10% FBS and 1% antibiotics) at a density of 10,000 cells for a live/dead cell assay, 50,000 cells for collagen 1 (COL1) staining per well on 8-well chamber slides (IWAKI, Sizuoka, Japan), and 50,000 cells for real-time PCR and alizarin red staining assays per well in 24-well plates.

### Live/dead cell assay

Twenty-four hours after cell seeding, the cells were evaluated using a live/dead staining assay according to the manufacturer’s protocol (Live-Dead Cell Staining Kit, Enzo Life Sciences, Farmingdale, NY, USA). Fluorescence images were quantified with an inverted fluorescence microscope (Olympus FSX-100). The cell quantities are expressed as the percentage of live-to-dead cells.

### Migration assay

A migration assay was performed using polyethylene terephthalate cell culture inserts with an 8-μm pore size (Millicell cell culture insert, Merck Millipore, MA, USA) in a 24-well plate. The lower compartment of each well was filled with medium supplemented with 20% conditioned media and 80% basic cell culture media. After starvation with DMEM containing 0.5% FBS for 12 h, 10,000 resuspended cells were seeded in the upper compartment. After 24 h of incubation, the cells were fixed with 4% formaldehyde for 2 min. Then, the cells were permeabilized with acetone for 15 min and stained with Giemsa solution for 20 min. The upper side of the filter membrane was rinsed and gently wiped with a cotton swab to remove cell debris. Images of the migrated cells were captured, and the number of cells under the filter was counted under a microscope (AXIO Zoom. V16, Carl Zeiss).

### Real-time PCR analysis

Total mRNA was isolated from RGFs at 3 and 7 days and from ROBs at 3 and 14 days poststimulation with conditioned medium to determine the relative mRNA levels of TGF-β, VEGF, and COL1 in the RGFs and runt-related transcription factor 2 (Runx2), COL1, and osteocalcin (OCN) in the ROBs. The specific time points of days 3 and 7 for RGFs, and days 3 and 14 for ROBs, were chosen based on the results from a similar previous study [11]. The sequences of primers used for the genes were generated according to the information presented in Table 1. mRNA isolation was performed using a ReliaPrep RNA Cell Miniprep system (Promega, Madison, WI, USA), and real-time PCR was performed using a GoScript reverse transcription system (Promega) and quantified on a StepOne Plus PCR system (Applied Biosystems, Waltham, MA, USA). The ∆∆Ct method was used to calculate gene expression levels normalized to the expression of β-actin.

**Table 1:** List of primer sequences for real-time PCR

Gene	Primer sequence (5′–3′)	
	Forward	Reverse
TGF-β	CGGCAGCTGTACATTGACTT	AGCGCACGATCATGTTGGAC
VEGF	CTACCTCCACCATGCCAAGT	CACACTCCAGGCTTTCATCA
COL1	ATGGATGAGGAAACTGGCAACT	GCCATCGACAAGAACAGTGTAAGT
Runx2	GCAGTTCCCAAGCATTTCATC	GTGTAAGTAAAGGTGGCTGGATA
OCN	GCTCAHCCTTCGTGTCCAAG	CCGTCGATCAGTTGGCGC
β-actin	GCTCGTCGTCGACAACGGCTC	CAAACATGATCTGGGTCATCTTCTC

TGF-β, transforming growth factor-β; VEGF, vascular endothelial growth factor; COL1, collagen I; Runx2, runt-related transcription factor 2; OCN, osteocalcin

### Collagen immunofluorescence staining

Fourteen days after seeding with conditioned medium, RGFs were investigated for COL1 expression by using immunofluorescence staining. The time point was chosen based on the previous work [11]. The RGFs were fixed with 4% formaldehyde for 10 min, permeabilized with PBS containing 0.2% Triton X-100, and blocked with PBS containing 1% bovine serum albumin (BSA; Sigma Aldrich) for 1 h. Subsequently, the cells were incubated overnight at 4 °C with an anti-mouse monoclonal COL1 antibody (MA1-26771; Invitrogen, Waltham, MA, USA) diluted 1:200 in PBS containing 1% BSA. After the cells were washed with PBS, they were incubated for 1 h at room temperature with secondary antibodies (goat anti-mouse Alexa Fluor Plus 488, Invitrogen) diluted 1:200 in PBS containing 1% BSA. Before visualization, the samples were mounted with VECTASHIELD containing DAPI nuclear stain (Vector, Burlingame, CA, USA). Images were captured with a fluorescence microscope (Olympus FSX-100). The optical density of fluorescently stained COL1 was quantified with ImageJ software (NIH, Bethesda, MD, USA) on the basis of the intensity of green staining using a color threshold that included parameters for hue, saturation and brightness.

### Alizarin staining

ROBs were cultured in osteogenic differentiation medium, which consisted of DMEM supplemented with 10% FBS, 1% antibiotics, 50 µg/mL ascorbic acid (Sigma Aldrich) and 10 mM β-glycerophosphate (Sigma Aldrich), to promote osteoblast differentiation. Alizarin red staining was performed to determine the presence of extracellular matrix mineralization. After 14 days of culture, the osteoblasts were fixed in 96% ethanol for 15 min and stained with 0.2% alizarin red solution (Sigma Aldrich) in water (pH 6.4) at room temperature for 1 h. Images were captured with a microscope (AXIO Zoom. V16), and the stained area was semiquantified with ImageJ software.

#### Statistical analysis

For each experiment, three independent experiments for each condition were performed with three or more replicates. The means and standard errors were calculated and analyzed for statistical significance through one-way analysis of variance with Tukey’s test for the live/dead assay, migration assay, COL1 staining, and alizarin red staining, and two-way analysis with the Sidak test was performed for real-time PCR analysis. All analyses were performed with GraphPad Prism 9 software (GraphPad Software, Inc., La Jolla, CA, USA), and * indicates a *p* value < 0.05, which was considered to indicate statistical significance.

## Results

### Characteristics of iPRF and iBMAC

The iBMAC was successfully separated into layers using the same centrifugation protocol used for iPRF (Fig. 1A, C). iPRF and iBMAC became hydrogels in the cell culture dishes within a few minutes after being transferred via syringes (Fig. 1B, D). Since some red blood cell layers were included in iPRF and iBMAC, the colors on both concentrates looked reddish; however, compared to iPRF, iBMAC was a darker brownish-red (Fig. 1A–D). Histological observation revealed that hematoxylin-stained nucleated cells were occasionally observed in iPRF and were more often and evenly located in iBMAC (Fig. 1E, F). There were more nucleated cells, especially with the aggregated cluster consisting of platelets, in iPRF (Fig. 1G) and with the platelets and/or adipose cells in iBMAC (Fig. 1H).

**Fig. 2:**
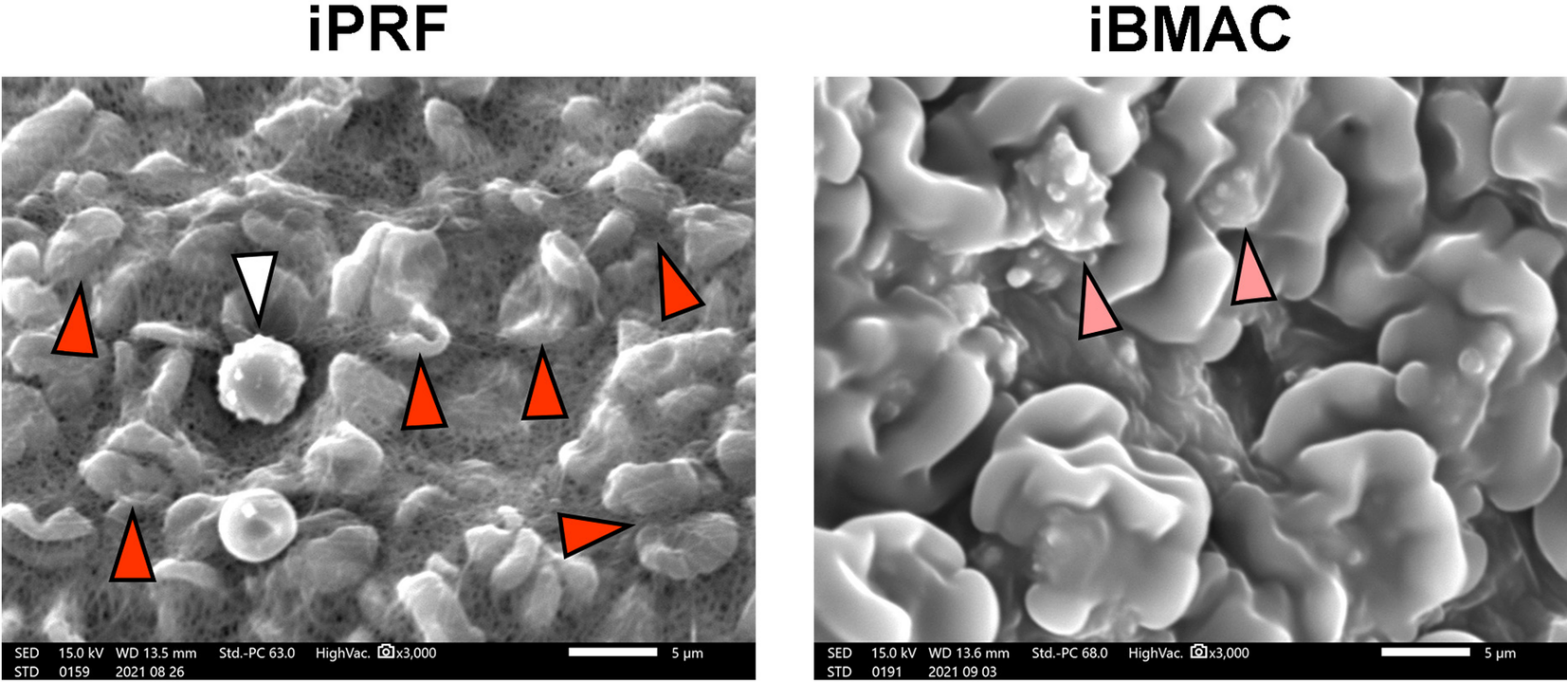
SEM images showing the concentrates on the surface of iPRF or iBMAC. The white arrowheads represent leukocytes, red arrowheads RBCs, and pink arrowheads indicate embedded leukocytes/bone marrow stromal cells. (Scale bar: 5 µm.)

SEM revealed red blood cells (RBCs), leukocytes, platelet aggregates and fibrin networks on the surface of the iPRF specimens (Fig. 2). The iBMAC included dense and smooth surfaces and some embedded bone marrow cells (Fig. 2).

### Influence of iPRF and iBMAC on gingival fibroblasts

**Fig. 3:**
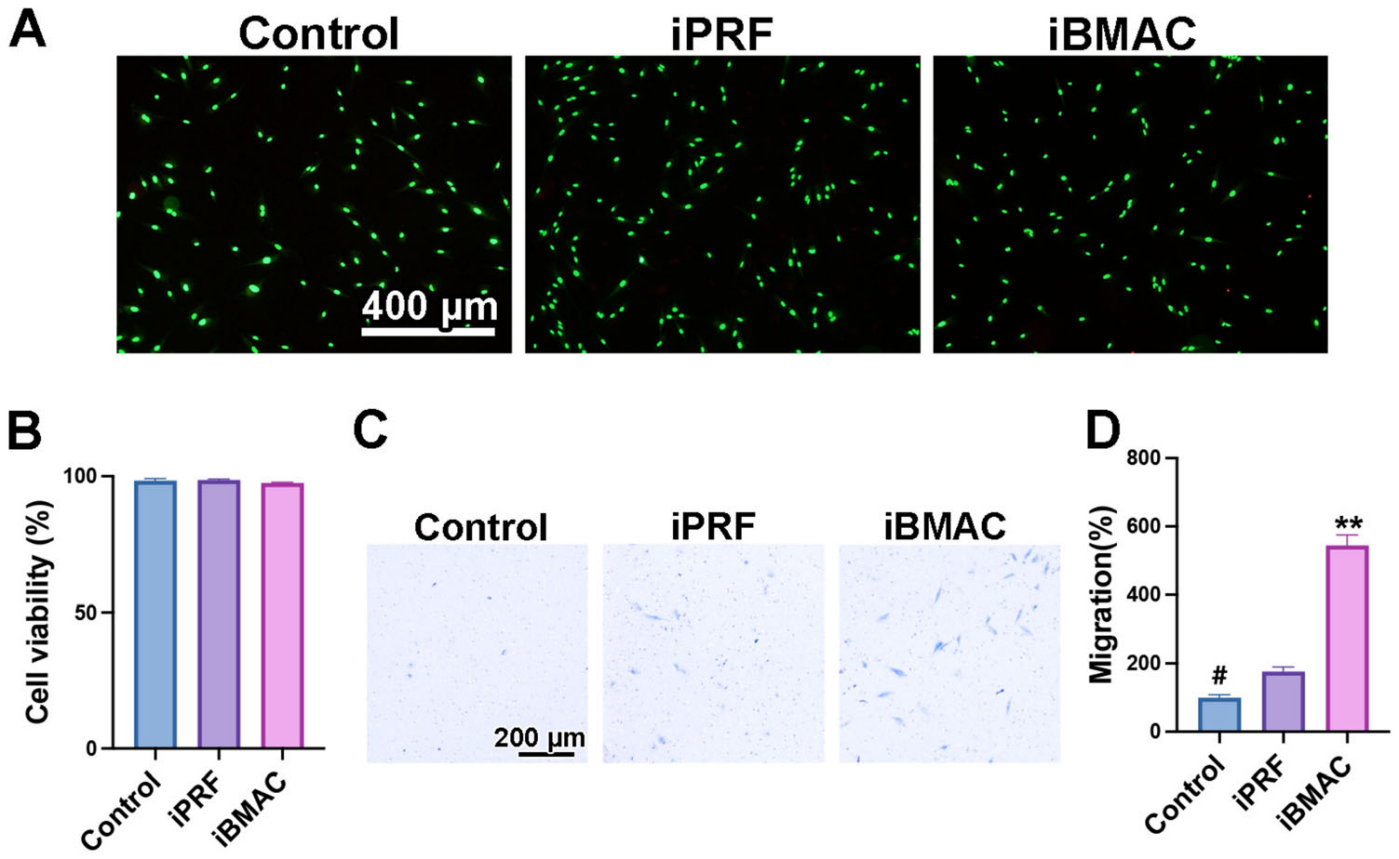
**A**, **B** Live/dead assay at 24 h after rabbit primary gingival fibroblasts (RGFs) were treated with control, iPRF or iBMAC. (**A**) Merged fluorescence images showing the live/dead staining assay, with viable cells appearing green and dead cells appearing red. (**B**) Cell viability was quantified as the percentage of living cells in each group. **C**, **D** Migratory ability of RGFs 24 h after each treatment. (**C**) Representative images of the migrated cells in each group. (**D**) Quantification of the number of migrated cells. (# denotes significantly lower than all other modalities, *p* < 0.05; and ** denotes values significantly greater than those of all other treatment modalities, *p* < 0.05)

The viability, migration potential, and gene expression of gingival fibroblasts were evaluated after stimulation with iPRF or iBMAC (Fig. 3). There were a high number of living cells (green cells) and few dead cells (red cells) observed upon live/dead staining in all the groups (Fig. 3A, B). Compared with the control group, the iPRF group exhibited greater migration potential; however, the iBMAC group exhibited the highest migratory potential among the tested groups (Fig. 3C, D).

**Fig. 4:**
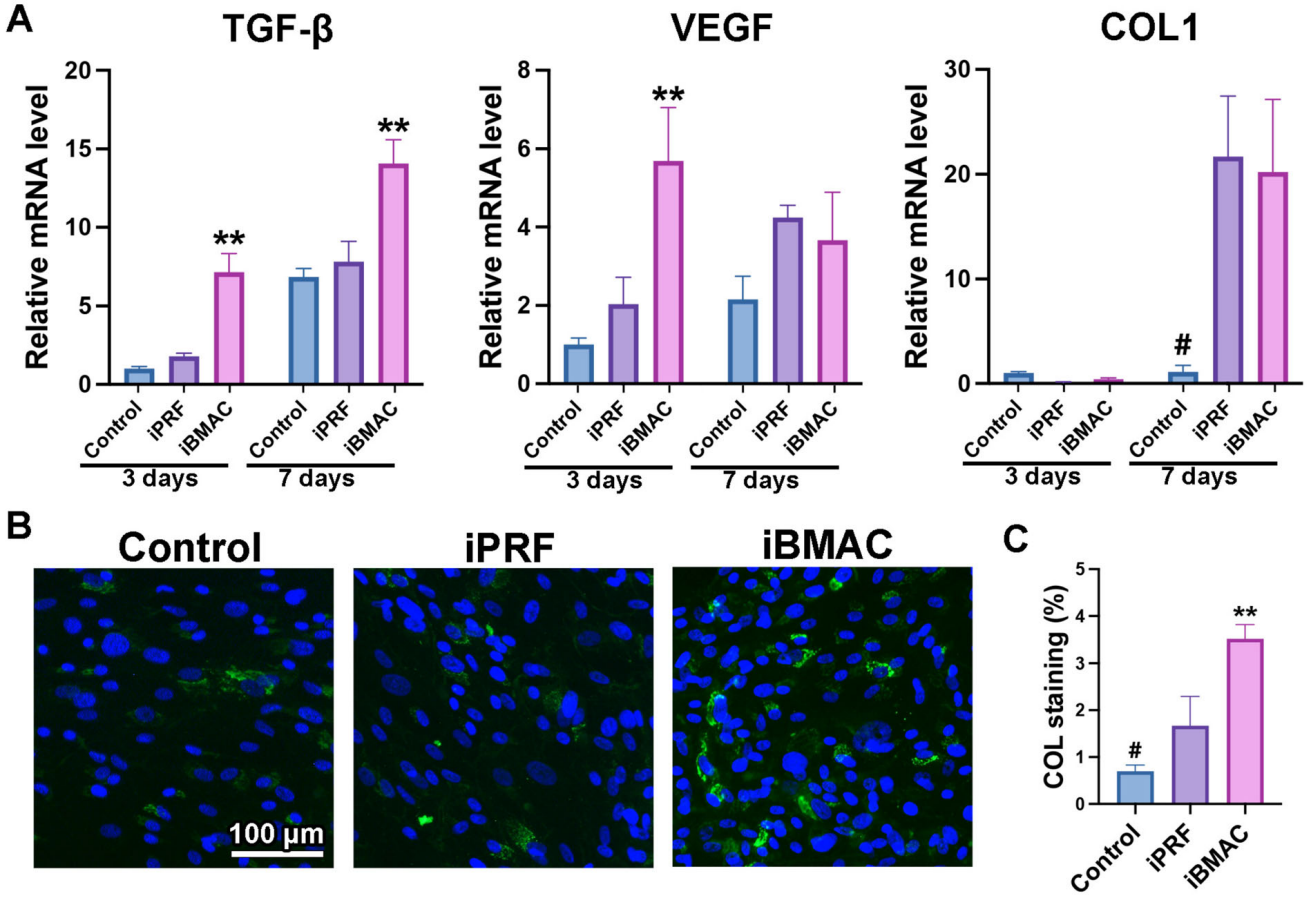
**A** Real-time PCR was performed to determine the relative mRNA levels of transforming growth factor-beta (TGF-β), vascular endothelial growth factor (VEGF) and collagen I (COL1) in RGFs cultured with control, iPRF, or iBMAC at 3 and 7 days postseeding. **B** Representative immunofluorescence staining of COL1 (green) with DAPI staining (blue) of RGFs at 14 days and **C** the quantified values of COL1 staining in comparison with each group. (# denotes significantly lower than all other modalities, *p* < 0.05; and ** denotes values significantly greater than those of all other treatment modalities, *p* < 0.05)

Real-time PCR analysis further revealed significant increases in the mRNA levels of TGF-β and VEGF in the iBMAC group compared with those in the control and iPRF groups on day 3 (Fig. 4A). Furthermore, the mRNA levels of COL1 were significantly greater in the iPRF and iBMAC groups than in the control group on day 7 (Fig. 4A). COL1 immunostaining of RGFs further revealed that COL1 synthesis was significantly promoted by iPRF and iBMAC compared to the control group, whereas iBMAC induced the highest COL1 expression among the groups (Fig. 4B, C).

### Influence of iPRF and iBMAC on osteoblasts

**Fig. 5:**
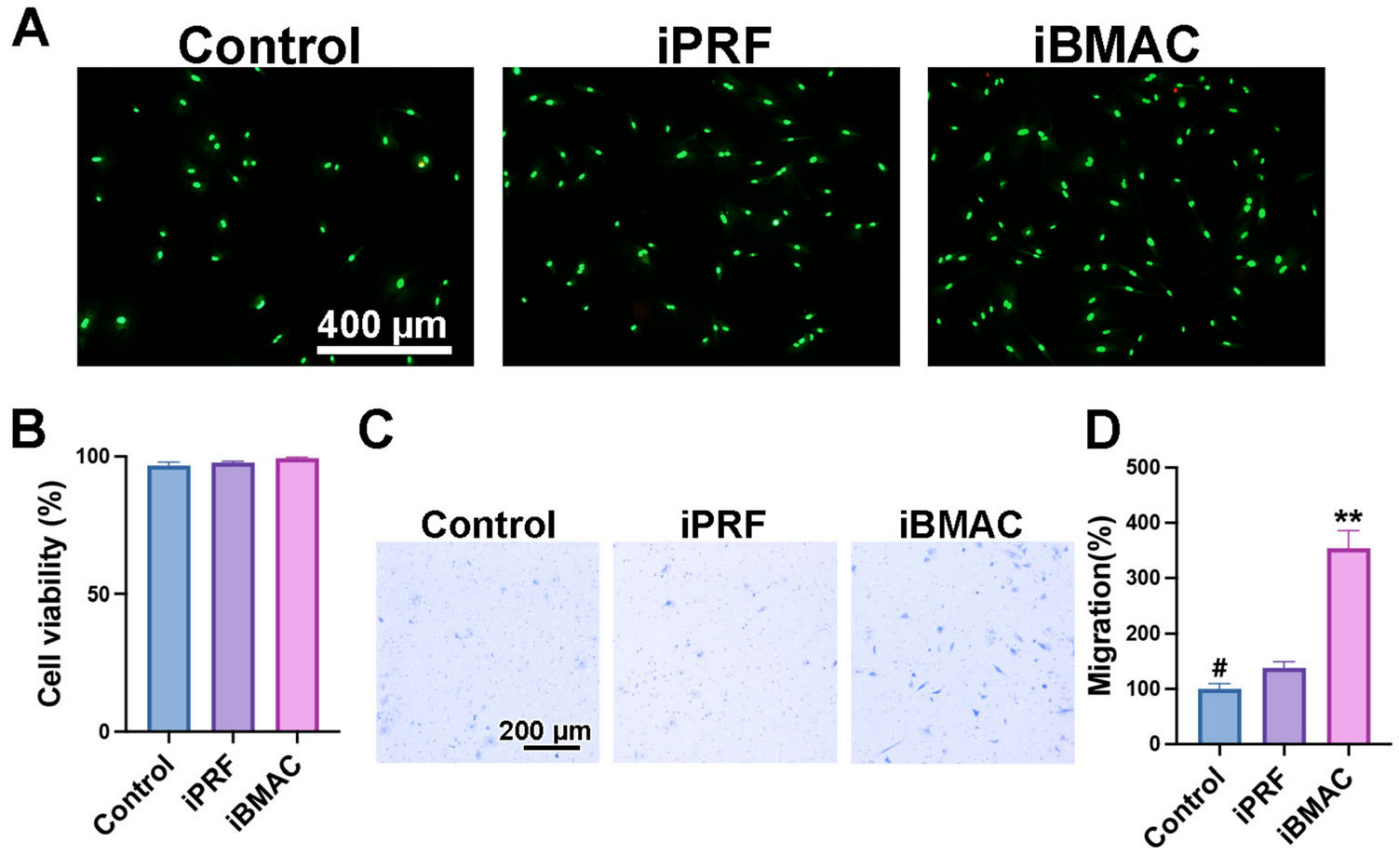
**A**, **B** Live/dead cell assay at 24 h after rabbit primary osteoblasts (ROBs) were treated with control, iPRF, or iBMAC. (**A**) Merged fluorescence images showing live/dead staining, with viable cells appearing green and dead cells appearing red. (**B**) Cell viability was quantified as the percentage of living cells in each group. **C**, **D** Migratory ability of ROBs at 24 h. (**C**) Representative image of the migrated cells in each group. (**D**) Quantification of the number of migrated cells. (# denotes significantly lower than all other modalities, *p* < 0.05; and ** denotes values significantly greater than those of all other treatment modalities, *p* < 0.05)

The viability, migration ability, and osteogenic potential of the ROBs after stimulation with iPRF or iBMAC were examined (Fig. 5). Similar to the RGFs, the ROBs exhibited excellent cell viability with almost 100% living cells in the live/dead assays after treatment with iPRF or iBMAC (Fig. 5A, B). Interestingly, greater migration potential was induced by iBMAC than the control or iPRF (Fig. 5C, D).

**Fig. 6:**
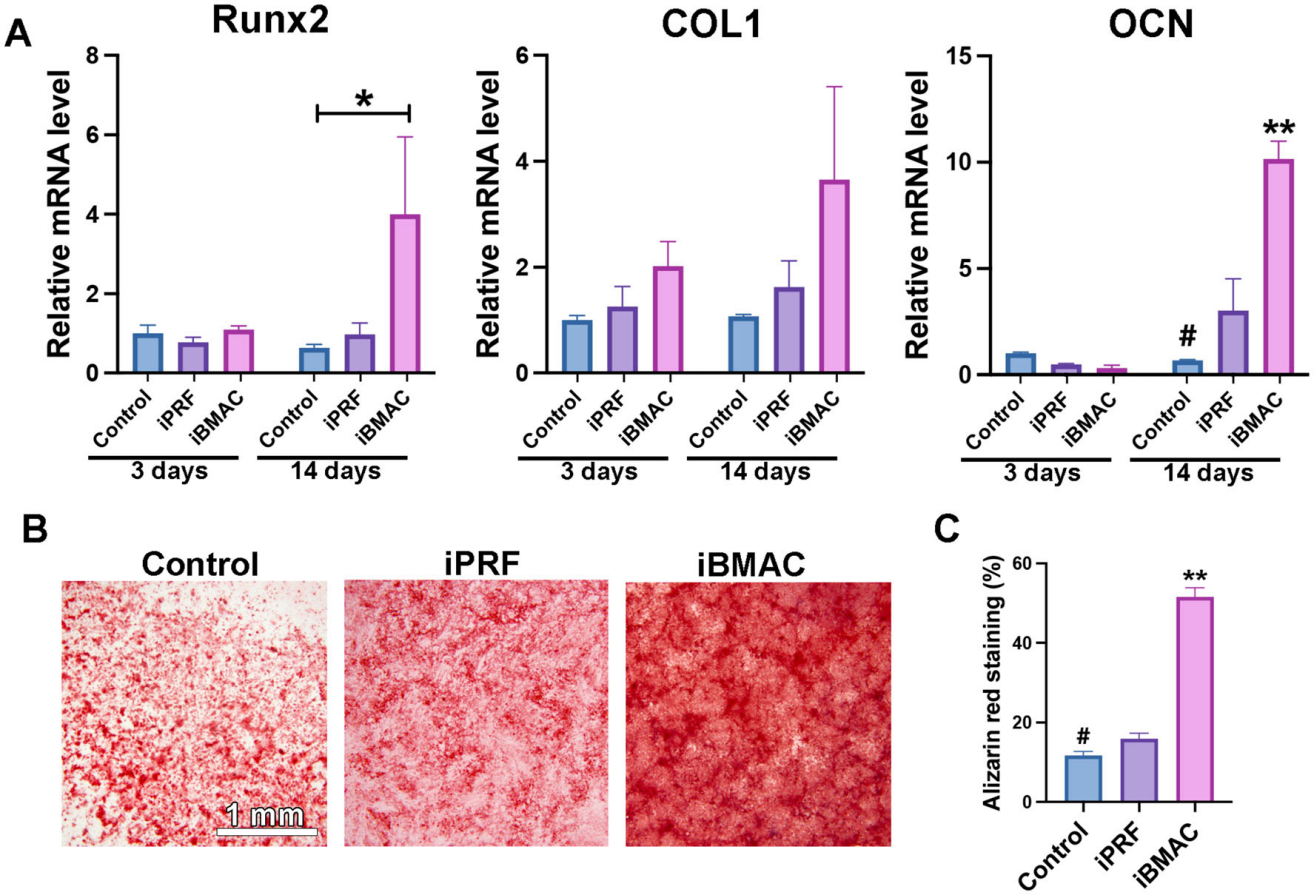
**A** Real-time PCR was performed to determine the relative mRNA levels of runt-related transcription factor 2 (Runx2), COL1 and osteocalcin (OCN) after 3 and 14 days of culture with control, iPRF or iBMAC in ROBs. **B** Representative alizarin red staining of ROBs 14 days after cell seeding and **C** the quantified values. (* denotes a statistically significant difference between groups, *p* < 0.05; # denotes a significantly lower value than that of all the other modalities, *p* < 0.05; and ** denotes a significantly greater value than that of all other treatment modalities, *p* < 0.05)

Real-time PCR analysis was performed to further evaluate the relative mRNA levels of osteoblast differentiation markers on ROBs. No significant differences were observed in the mRNA levels of COL1 on days 3 or 14 (Fig. 6A). However, compared with control treatment and iPRF treatment, iBMAC treatment significantly promoted OCN expression on day 14 (Fig. 6A). Alizarin red staining confirmed that mineralization potential was greater in the iBMAC group than in the other groups (Fig. 6B, C).

## Discussion

Conventional PRF does not attractively promote bone regeneration, unlike soft tissue healing [17, 18]. Nevertheless, using rabbit animal models, we previously developed a solid-type BMAC (sBMAC) that increased soft tissue healing and showed greater osteogenic capacity than that of PRF clots *in vitro* and *in vivo* [11, 12]. Thus, in the present study, we focused on the liquid form of BMAC and demonstrated that, compared with iPRF, iBMAC promoted osteoblast differentiation and gingival fibroblast activation; this effect most likely resulted from the abundant natural growth factors produced by the enriched bone marrow-derived stem cells within the iBMAC.

iPRF has been utilized for a variety of regenerative procedures, including the repair of articular joints, osteoarthritis management, treatment for temporal-mandibular joint disorders and combination therapy with various bone grafting materials or collagen membranes for bone regeneration [4, 19]. The liquid formulation of PRF generally remains liquid for approximately 15 min and can be utilized as an injectable biomaterial during this time [19]. Due to the presence of megakaryocytes, platelets and coagulation factors, the iBMAC may coagulate as well as the peripheral blood [20, 21]. The iBMAC fabricated in the present study exhibited characteristics akin to those of iPRF, as the iBMAC coagulates within 10–15 min (Fig. 1). Therefore, procedures analogous to those utilized for iPRF may be beneficial for the clinical application of iBMAC.

The cell composition of iPRF has been previously investigated by other groups. Notably, Miron et al. conducted a CBC analysis of human iPRF, reporting a 2.07-fold increase in platelet concentrations and a 23% increase in leukocytes when following the original protocol of 800 rpm for 3 min [15]. Compared with iPRF, which contains some leukocytes and platelets, iBMAC contains denser fibrin networks with significantly more enriched cells in bone marrow tissues, although accurate cell phenotypes in iBMAC remain unclear because structural observation of the cells was limited in the present study. (Figs. 1, 2). Furthermore, finding appropriate antibodies to detect cell markers in rabbit specimens is challenging, therefore, could not show any original cell characterization in this study. Nevertheless, conventional BMAC, which is produced with containing anticoagulants in a plastic tube, has also been investigated and found that conventional BMAC contains a heterogeneous agglomeration of numerous cell types, different mononuclear cell types including macrophages, lymphocytes, mast cells, and other cells, and not the mesenchymal cell lineage [22–24]. The standardization of iPRF and iBMAC, particularly in human samples, is necessary for future research.

Compared with the control, iBMAC treatment resulted in greater cell migration, angiogenesis, collagen synthesis, and osteoblast differentiation potential (Figs. 3, 4, 5, 6). Hematopoietic stem cells and mesenchymal stem cells are present within the bone marrow, albeit at low concentrations [23, 24]. It was reported that, compared with unprocessed bone marrow aspirate, conventional BMAC (prepared with anticoagulants) markedly amplifies the concentration of mesenchymal stem cell populations (approximately by a factor of four), platelets, and growth factors [25, 26]. The growth factors released from BMAC include TGF-β, PDGF, bone morphogenetic protein (BMP)-2, BMP-7, and FGF-2, which are important due to their anabolic and anti-inflammatory implications in tissue engineering [27, 28]. The concentration of FGF-2 was higher in BMAC than in PRP, whereas no significant differences in the levels of PDGF-BB, VEGF, TGF-β1, and BMP-2 were observed [29]. In earlier work, we investigated the levels of TGF-β, ALP, and OCN as markers of osteogenesis in the conditioned media from solid-type PRF, and all tested growth factors from BMAC demonstrated significantly higher release for 24 h when compared to PRF [30]. The solid-type PRF and BMAC were coagulated in glass tubes, whereas iPRF and iBMAC were coagulated after centrifugation and collecting from plastic tubes. Despite these differences in handling, the cellular components and growth factor release behavior should be comparable. Nevertheless, a direct comparison of growth factor release between iPRF and iBMAC would indeed be of great interest and is an important area for future research, particularly in large animals and humans.

Matrix metalloproteinases (MMPs) are crucial factors involved in tissue remodeling during various physiological and pathological processes, including morphogenesis, angiogenesis, tissue repair, cirrhosis, arthritis, and metastasis [31]. Platelet concentrates are known to show both anabolic effects and inhibitory actions on catabolic mediators released during the inflammatory cascade [32]. Previous studies have reported that PRP contains significantly higher concentrations of MMP-2, MMP-3, and MMP-12 compared to BMAC [32]. Additionally, it was reported PRF extracts have been shown to contain MMP-2 and MMP-9, suggesting that these factors contribute to its tissue regenerative capabilities [33]. The secretion behavior and functions of MMPs during tissue regeneration are not clear in the *in vitro* present study which did not consider any inflammation surroundings. Nevertheless, the presence of MMPs in iBMAC could contribute to enhanced tissue remodeling and repair, further supporting its therapeutic potential.

In general, an injectable material is easy to handle for closed wounds and can be combined with other biomaterials. iBMAC can potentially serve as an alternative to bovine-derived fibrin, functioning as a naturally sourced autologous fibrin matrix [4]. Furthermore, natural fibrin glue encompasses various bone marrow-derived growth factors, which can enhance angiogenesis and tissue regeneration within defects. The combination of iBMAC with other biomolecules, cells and/or growth factors could offer many additional advantages as a carrier system for tissue regeneration purposes [4].

Previous clinical reports in the oral surgery field presented the possibility of using BMAC with scaffolds such as collagen sponges, autogenous bone or PRF clots for bone regeneration surgery. Melville et al. [34] reported a successful case series of benign tumor mandibular reconstructions using freeze-dried cortical and cancellous bone combined with recombinant human BMP-2 and an absorbable collagen sponge as well as BMAC without any autogenous bone harvesting. The combination therapy showed advantages, namely, no donor site morbidity, a shorter intraoperative time, fewer admission days, and lower total costs [34]. Additionally, the findings from a randomized clinical trial conducted by Fontes Martins et al. elucidated that the application of BMAC integrated into PRF plugs enhances the augmentation of extraction sockets [35]. The results indicated that the quantity of mineralized tissue formation was significantly greater than that in the control cohort following a healing period of six months, with an increase of 10% over the assembly treated solely with PRF [35]. Combination therapies using iBMAC, similar to BMAC, may offer promising alternatives for bone reconstruction protocols in the field of oral and maxillofacial surgery.

While our findings offer valuable insights into the use of iBMAC for the regeneration of gingival tissues and bone tissues, there may be several limitations regarding the experimental approaches used. In the present study, peripheral blood and bone marrow aspirates were obtained from rabbits due to the feasibility of collecting adequate fluid volumes from the same individual specimens simultaneously, as previously reported [11, 12]. The distinctions between humans and rabbits regarding cellular and molecular characteristics in blood/bone marrow fluid must be considered. Additionally, it is essential to consider the discrepancies between *in vitro* experimental findings and *in vivo* preclinical studies when evaluating tissue responses to iBMAC.

In conclusion, compared with iPRF, iBMAC treatment led to greater cell migration, angiogenesis, and collagen synthesis and greater osteoblast differentiation potential in gingival fibroblasts and osteoblasts. Therefore, iBMAC might be a new candidate for promoting wound healing and bone regeneration. Since iPRF does not promote bone regeneration, iBMAC might be desirable for bone regeneration procedures in certain cases. Nevertheless, further preclinical and clinical studies are needed to investigate the functions of iBMAC in soft and bone tissues in the body.

## Data Availability

The datasets used and/or analyzed during the present study are available from the corresponding author on reasonable request.
